# Association of Interleukin-1α with periodontitis among Indians: a narrative review

**DOI:** 10.3205/dgkh000525

**Published:** 2024-12-16

**Authors:** Urvi Vashistha, Nitik Baisoya, Pranav Bansal, Pranav Trishal, Ruchi Pandey

**Affiliations:** 1 Manav Rachna Dental College, School of Dental Sciences, MRIIRS, Faridabad, Haryana, India; 2 Department of Periodontology, Manav Rachna Dental College, School of Dental Sciences, MRIIRS, Faridabad, Haryana, India

**Keywords:** periodontitis, Indian, IL-1 alpha polymorphism

## Abstract

**Background:**

The etiology of periodontitis is multifactorial, involving interactions between bacterial pathogens, host immune response, and environmental factors. Among the host immune factors, interleukin-1 alpha (IL-1α) has been implicated in the pathogenesis of periodontitis. Many studies have aimed to find the association between IL-1α and periodontitis in various populations worldwide. However, the evidence in the Indian population is limited. Therefore, this study aims to analyse data from the literature related to the genetic correlation between IL-1α polymorphisms and periodontitis among Indians.

**Method:**

Only case-control and cross-sectional studies investigating the association between IL-1α polymorphisms (+4,845 and –889) and various forms of periodontitis in the Indian population were included. PubMed, Medline, Web of Science, Cochrane based reviews, Scopus, and Google Scholar were used for the search.

**Results:**

The findings demonstrate a mixed pattern of associations between these polymorphisms and periodontitis across different regions of India.

**Conclusion:**

The correlation of periodontitis with IL-1α polymorphism in Indians lacks evidence.

## Zusammenfassung

**Hintergrund:**

Die Ätiologie der Parodontitis ist multifaktoriell und beinhaltet Wechselwirkungen zwischen bakteriellen Erregern, Immunantwort des Wirts und Umweltfaktoren. Unter den Wirtsimmunfaktoren wurde Interleukin-1 alpha (IL-1α) mit der Pathogenese der Parodontitis in Verbindung gebracht. Viele Studien haben versucht, den Zusammenhang zwischen IL-1α und Parodontitis in verschiedenen Bevölkerungsgruppen weltweit zu ermitteln. In der indischen Bevölkerung sind die Erkenntnisse jedoch begrenzt. Daher zielt die Studie darauf ab, Daten über die genetische Assoziation zwischen IL-1α Polymorphismen und Parodontitis bei Indern zu analysieren. Ziel der Untersuchung war die Analyse von Forschungsdaten über den Zusammenhang zwischen IL-1α Polymorphismen (+4.845 und –889) und verschiedenen Formen der Parodontitis in der indischen Bevölkerung

**Methode:**

Es wurden nur englischsprachige Fall-Kontroll- und Querschnittsstudien aus Indien berücksichtigt, die sich mit dem Zusammenhang zwischen IL-1α und Parodontitis befassen. Für die Suche wurden PubMed, Medline, Web of Science, Cochrane-basierte Reviews, Scopus und Google Scholar verwendet.

**Ergebnisse:**

Die Ergebnisse zeigen ein gemischtes Muster von Assoziationen zwischen diesem Polymorphismus und Parodontitis in verschiedenen Regionen Indiens.

**Schlussfolgerung:**

Der Zusammenhang zwischen Parodontitis und IL-1α-Polymorphismus ist bei Indern nicht belegt.

## Introduction

Periodontitis is governed by the environment, local factors, genetic makeup and phenotype of an individual [1]. The pervasiveness of periodontitis in Indians averages between 63% to 89% across different age groups [2]. Immunity plays a decisive role in maintaining homeostat is during bacterial infection in periodontitis [3]. The predominant cytokines released belong to interleukins, mainly Interleukin-1 alpha (IL-1α) and Interleukin 1β (IL-1β). 

The expression of IL-1α is influenced by a gene located on chromosome 2q12-21; its locus is at –889 and +4845 [4]. IL-1α is a cytokine primarily secreted by activated resident gingival cells in response to bacterial challenges [5]. It contributes to the devastation of periodontal tissues by playing a critical part in the initiation and continuation of the inflammatory response. It has been demonstrated that IL-1α increases the expression of other inflammatory mediators, including prostaglandins and chemokines, and stimulates the synthesis of matrix metalloproteinases directly influencing the breakdown of extracellular matrix [6]. Moreover, IL-1α initiates osteoclastic pursuit, thereby increasing bone destruction [7]. 

Understanding the function of IL-1α in the pathogenesis of periodontitis among the diverse Indian population is critical, since polymorphism can increase the prevalence of disease under disease-promoting environmental conditions, thereby governing the expression of IL1α. However, the research done on the Indian population is limited.

The incidence of periodontitis differs in India throughout socioeconomic categories and geographical areas, indicating the possible impact of both environmental and genetic variables on disease susceptibility [2]. Furthermore, the genetic diversity of the Indian population may influence the production and control of inflammatory mediators such as IL-1α [8].

The aim of the current systematic review was to find the factors associated with IL-1α in causing periodontitis among Indians. 

## Method

Case control and cross-sectional Indian studies on the association of IL-1α with periodontitis, available as full-length articles in English, were included. Reviews, case reports, and editorials were excluded. An extensive literature search was carried out on electronic databases, i.e., PubMed, Medline, Web of Science, Cochrane based reviews, Scopus, and Google Scholar. Medical Subject Headings (MeSH) terminology and pertinent keywords were combined in the search strategy, included “interleukin-1 alpha”, “IL-1α”, “polymorphism”, “periodontitis”, “chronic periodontitis”, “aggressive periodontitis”, and “India” or “Indian population”. Eligible studies were retrieved and assessed for inclusion by the same two independent reviewers. The review is registered in open science framework and can be cited using osf.iordek/5. To gather pertinent data from the included studies, a standard data extraction form was employed. The following information was extracted:

- Study characteristics (author, study design, year of publication, and Indian area)
- Participant attributes (gender, age, and sample size) (see Table 1 (Tab. 1))

## Results

The included studies were conducted across different regions of India. A total of 1,080 subjects were studied, including periodontally affected as well as periodontally healthy individuals. The participants in the included studies were adults of varying age groups, with a mean age of 42 years (Table 1 (Tab. 1)).

Two studies reported a positive association between the IL-1α (+4,845) polymorphism and periodontitis [9], [10], whereas the other studies showed a negative association [11], [12]. Two studies investigated the IL-1α (–889) polymorphism and found a significant correlation [13], [14] (Table 1 (Tab. 1)). 

## Discussion

The link between IL-1α polymorphisms and periodontitis in the Indian population remains complex and heterogeneous, influenced by factors such as genetic diversity, environmental exposures, and disease subtypes. Several studies highlighted the presence of distinct genetic subgroups within the Indian population, influenced by factors such as geographic location, endogamy, and historical migration patterns [15]. 

This genetic heterogeneity established through various GWAS studies could potentially influence the distribution and frequencies of IL-1a polymorphisms, as well as their functional implications in the pathogenesis of periodontitis for e.g. in the study by Munz et al. where in a pooled data, a locus at SIGLEC5 (sialic acid binding Ig-like lectin 5) and a chromosomal area downstream of the DEFA1A3 locus (defensin alpha 1–3) were linked to both forms of disease characteristics and were significantly related with periodontitis at the genome-wide level and other similar studies [16], [17], [18], [19], [20], [21], [22].

Environmental factors, including smoking, diet, and oral hygiene practices, are well-established risk factors for periodontitis [23]. These factors may also modulate the expression and activity of IL-1α, thereby influencing its role in the disease process. The included studies did not consistently report on environmental exposures of the study participants, which could contribute to the heterogeneity found. Future studies should consider incorporating environmental factors as potential confounders or affect modifiers in their analyses.

The association between IL-1α polymorphisms and periodontitis subtypes, such as chronic and aggressive forms, was explored in some of the included studies (Table 1 (Tab. 1)). The pathogenesis and immunological mechanisms underlying these subtypes may differ, potentially influencing the role of IL-1α in the disease process influenced by genotype [24]. For instance, aggressive periodontitis is thought to have a stronger genetic component than does chronic periodontitis [25]. Varying associations observed between IL-1α polymorphisms and periodontitis subtypes could reflect these underlying differences in disease mechanisms.

## Limitations and future directions

This study had certain limitations, including the potential for publication bias and the moderate to high heterogeneity observed among the included studies. Future investigations should focus on addressing these limitations by conducting more comprehensive, well-designed studies with standardized methodologies, encompassing reporting of participant characteristics, environmental exposures, and genetic data.

## Conclusion

The current systematic review failed to find an association of IL-1α polymorphism with periodontitis due to paucity of supporting studies in literature. Nevertheless, despite the heterogeneity of the findings, the identification of specific IL-1α polymorphisms associated with periodontitis in the Indian population could have potential clinical implications. These polymorphisms could serve as potential genetic markers for risk assessment and early disease detection, particularly in high-risk individuals or populations [26]. Furthermore, understanding the involvement of IL-1α in the pathologic process of periodontitis could guide the development of targeted therapeutic interventions, such as anti-inflammatory agents or personalized treatment strategies tailored to the genetic profiles of individuals or specific population groups [27].

## Notes

### Competing interests

The authors declare that they have no competing interests.

## Figures and Tables

**Table 1:**
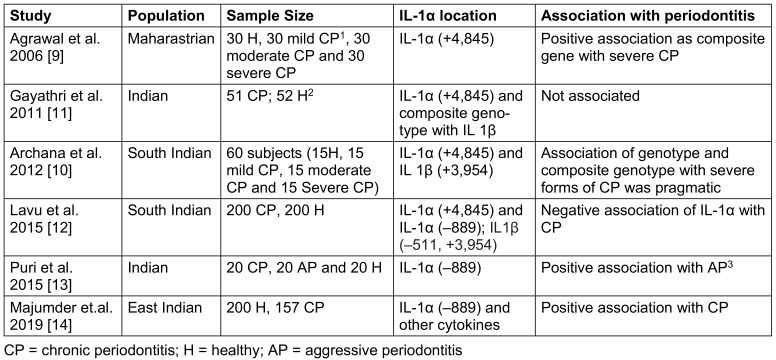
Association of IL-1α with demographics, disease cases and single nucleotide polymorphisms (SNPs)
